# Editorial: The Complex Biopsychosocial Interactions That Create Stress Resilience

**DOI:** 10.3389/fnbeh.2021.795312

**Published:** 2021-11-22

**Authors:** Deborah Suchecki, Juliana Nery Souza-Talarico, Clement Hamani, Jocelien D. Olivier

**Affiliations:** ^1^Departamento de Psicobiologia, Escola Paulista de Medicina, Universidade Federal de São Paulo, São Paulo, Brazil; ^2^College of Nursing, The University of Iowa, Iowa City, IA, United States; ^3^Faculdade de Enfermagem Universidade de São Paulo, São Paulo, Brazil; ^4^Sunnybrook Research Institute, University of Toronto, Toronto, ON, Canada; ^5^Groningen Institute for Evolutionary Life Sciences, University of Groningen, Groningen, Netherlands

**Keywords:** network analysis, coping strategies, sense of coherence, social support, biological factors, psychological factors, animal models, human population

Stress is present in our daily surrounding. Although it is essential for survival and functioning, it may become pathological when coping strategies are dysfunctional. While some people are vulnerable, others are more resistant to stressors. Although individual variability in stress resilience has been well-characterized, its underlying mechanisms remain elusive. Resilience is a dynamic process related to the individual's capacity to adaptively cope with adversity, trauma, threats or significant stressors. While a small proportion of individuals succumb to traumatic events or chronic stress and develop stress-related psychiatric disorders, most people are not severely affected and some may even flourish in adverse or stressful circumstances. Biologic, psychological and social factors interact with each other creating a physiologic and behavioral repertoire, which may enable individuals to thrive in face of adversity.

At present, stress resilience and coping strategies are important tools for the treatment of psychiatric diseases. In this Research Topic entitled “The Complex Biopsychosocial Interactions that Create stress Resilience” we have brought together the most recent research findings in resilience to stress. Original and review papers on pre-clinical models and human populations present different aspects of what is collectively known as resilience. The volume is organized in four themes: Resilience after stressful experiences, Social effects on resilience, Positive effects on stress resilience and Effects of resilience in professional caregivers.

The review by Matheson et al. presents concepts related to psychosocial factors of resilience and the importance of coping strategy, social support and spirituality in dealing with different scenarios of social adversity, such as psychological abuse in intimate relationships, political, ethnic and religious refugees, and indigenous peoples. Traumatic events early in life may represent a risk factor for the development of emotional psychiatric disorders. Evaluating 170 community-dwelling adults, Seok et al. investigated the interaction between early trauma (e.g., abuse and neglect during childhood) and recent stressful events on depression, anxiety and anger symptoms at adulthood. Although recent stressors increase anxiety, the interaction of early life trauma and recent stressors potentiate depression and anger scores. To investigate the impact of early life adversity on an elder population, Thoma et al. assessed victims of a wide range of childhood adversity (risk group) and an age-matched control group for their resilience resources and self-esteem. They used a network analysis of specific resilience factors and showed that both the risk and control groups demonstrated positive resilience resource interrelations. This may be useful for the development of potential clinical intervention targets in adult or elderly survivors of early life adversity. Besides early life adversity, natural disasters are likely to result in emotional disorders, including depression and posttraumatic stress disorder (PTSD). The study by Rossi et al. with university students who survived an earthquake in L'Aquila, Italy, showed that exposure, material loss or psychological suffering associated with the event did not affect the students in any major way. This, however, was reported in individuals with a high perception of self.

Social animals and humans show better recovery from stressful experiences when in groups. Two studies in this Research Topic have focussed on social aspects, cognition and stress coping. Souza-Talarico et al. investigated the role of social support in a population-based cohort of older adults, showing that high levels of family and community support increased cognitive performance. Emotional components of social support (e.g., loving and empathy) were critical in the relationship between social interactions and cognitive health (Souza-Talarico et al.). The impact of social relationships on general health can be tested in pre-clinical studies using social isolation or individual housing. Using this paradigm in an animal model of breast cancer, Berry et al. demonstrated that social isolation increased corticosterone levels, decreased active coping behavior and gene expression of neuropeptide Y, and other molecular elements related to metabolism. In addition, social isolation increased the vulnerability of female mice to maladaptive behaviors, accelerated the development of breast cancer and impaired the regulation of resilience factors.

Besides social buffering, other factors also positively contribute to resilience. Roversi et al. used a tactile stimulation approach early in life in a genetic model of anxiety and depression to explore the effects of beneficial stimuli. Serotonin transporter (5-HTT) heterozygous knockout (5-HTT+/-) rats serve as a model of the human short polymorphism of the 5-HTT, known to be more vulnerable to stress (Houwing et al.). Mimicking maternal licking and grooming, tactile stimulation administered in 5-HTT+/- offspring was shown to improve social and affective behaviors and alter molecular parameters related to glucocorticoid, GABA and glutamate functioning. Ketamine, an NMDA receptor antagonist, modulates the glutamatergic system, and is currently being used in several stress-related and alcohol use disorders. The review by Strong and Kabbaj provides evidence of the effectiveness of ketamine treatment on neuroplasticity induced by chronic alcohol use. In a narrative review, Arida and Teixeira-Machado polled 18 studies to summarize the benefits of aerobic fitness to brain resilience from early stages of life to the elderly. Exercise was found to enhance cognitive capacity of the offspring by increasing postnatal neurogenesis and neuronal growth. During adulthood and aging, aerobic exercise protects individuals from cognitive decline and depression by reducing neuroinflammation and increasing neuroplasticity, angiogenesis, and brain vasculature. Brain derived neurotrophic factor has been suggested to mediate those positive effects by counterbalancing the negative stress consequences.

Providing health care may come with a risk for professional caregivers, which are often faced with stress as part of their job. Dealing with psychiatric patients exposed to trauma may sometimes personally expose healthcare workers to harm (e.g., verbal and physical aggression). This, along with the heavy workload and unpredictable schedules, may lead to stress-related disorders. Indeed, health care professionals are more likely to develop burnout syndrome than the general population. In a study with Chinese nurses, Song et al. explored the mediating role of sleep on perceived stress and job burnout. They report that stress has a direct impact on burnout leading to poor quality of sleep. The authors propose that interventions and strategies that improve sleep quality and stress coping may improve health in general. Stress resilience in health care professionals may be a buffer for stress-related problems and increase job satisfaction. Bürgin et al. investigated the sense of coherence (SoC), self-efficacy, self-care and measured stress markers in youth residential caregivers. SoC and self-care were both associated with lower cortisol: DHEA (dehydroepiandrosterone) ratio implying that youth residential institutions may want to provide programs that enhance SoC and self-care practices to improve the health of caregivers. The results presented in this Research Topic demonstrate a crucial role for biopsychosocial interactions in the resilience for stress and proposes several indicators to create stress resilience.

## Author Contributions

DS, JS-T, CH, and JO contributed to the writing and editing of the text. All authors read and approved the final version of the manuscript.

## Conflict of Interest

The authors declare that the research was conducted in the absence of any commercial or financial relationships that could be construed as a potential conflict of interest.

## Publisher's Note

All claims expressed in this article are solely those of the authors and do not necessarily represent those of their affiliated organizations, or those of the publisher, the editors and the reviewers. Any product that may be evaluated in this article, or claim that may be made by its manufacturer, is not guaranteed or endorsed by the publisher.

